# The Complexity of Managing a Burned Irreducible Umbilical Hernia in an Adult

**DOI:** 10.7759/cureus.14030

**Published:** 2021-03-22

**Authors:** Samuel Teklay, Edward Balai, Lopa Patel

**Affiliations:** 1 Plastic Surgery, Queen Elizabeth Hospital, Birmingham, GBR; 2 Plastic Surgery, Sandwell and West Birmingham Hospitals NHS Trust, Birmingham, GBR

**Keywords:** burn injury, abdomen ventral hernia

## Abstract

We present a case of a 65-year-old male with a longstanding non-symptomatic irreducible umbilical hernia who presented with a three-day-old full-thickness thermal burn to the hernia. The burn was sustained while operating a metal drop forger, where the patient was repeatedly exposed to 30-second bursts of heat from the furnace, with temperature exceeding 1350°C. He estimated he had this exposure approximately 48 times during an 8-hour shift, giving him a total of 24 minutes cumulative exposure to the heat. The patient reported that he normally wore an abdominal binder under his heat-resistant apron to temporarily flatten and protect his hernia. On the day of the injury, he had not been wearing this binder. The patient was initially unaware that he had sustained a burn; upon delayed presentation to the hospital, he had cellulitis surrounding a 0.25% total body surface area (TBSA) full-thickness burn. Contrast-enhanced CT abdomen demonstrated an umbilical hernia with a neck diameter of 2.3cm, with breach of the hernia fascia but no communication between the bowel and burnt tissue. After discussion between Plastic Surgery and General Surgery teams the decision was made to manage the burn non-operatively with daily flamazine dressings and empirical antibiotics for the cellulitis. Once this area had healed, elective mesh repair of the umbilical hernia was carried out. This is the first adult case of a full-thickness burn overlying an umbilical hernia to be reported in the literature. The case highlights both an unusual aetiology and a rare injury, as well as the multi-disciplinary teamwork required to manage it successfully.

## Introduction

The aetiology of burns is most often thermal (flame or scald), chemical or electrical [1]. Burns are often accidental and can be an occupational hazard despite employers adhering to the legal requirements of health and safety. They can be defined as superficial, partial thickness or full thickness depending on the depth of tissue injury. We present a unique case of a burn involving an umbilical hernia due to an unusual occupational aetiology. Burns involving the skin over an umbilical hernia are very rare, to our knowledge, this is the first time a case of an adult with this type of injury has been reported in the literature.

## Case presentation

A 65-year-old diabetic male was referred to our burns centre from the accident and emergency department of a peripheral hospital with a full-thickness burn to his longstanding, irreducible umbilical hernia. This injury was sustained whilst at work where he operated as a metal drop forger, shaping metal bars under intense furnace heat. When placing metal bars into the furnace he was exposed to temperatures exceeding 1350°C for 20 to 30 seconds a time approximately 48 times a day giving him a total of 24 minutes exposure in an eight-hour shift. 

Health and safety regulations on site stipulate a heat-resistant apron must be worn which the patient was wearing at the time of injury. However due to his umbilical hernia he also normally wore a waist support belt which temporarily flattened the hernia and reduced the risk of this protruding aspect of the abdomen getting injured. On the day of the incident, the patient was not wearing this protective belt. There was a delay in the patient presenting to the hospital as he was initially unaware that the injury had occurred, presenting on day 3 post-injury when he noticed a discharge from this area of skin was making the front of his shirt wet.

**Figure 1:**
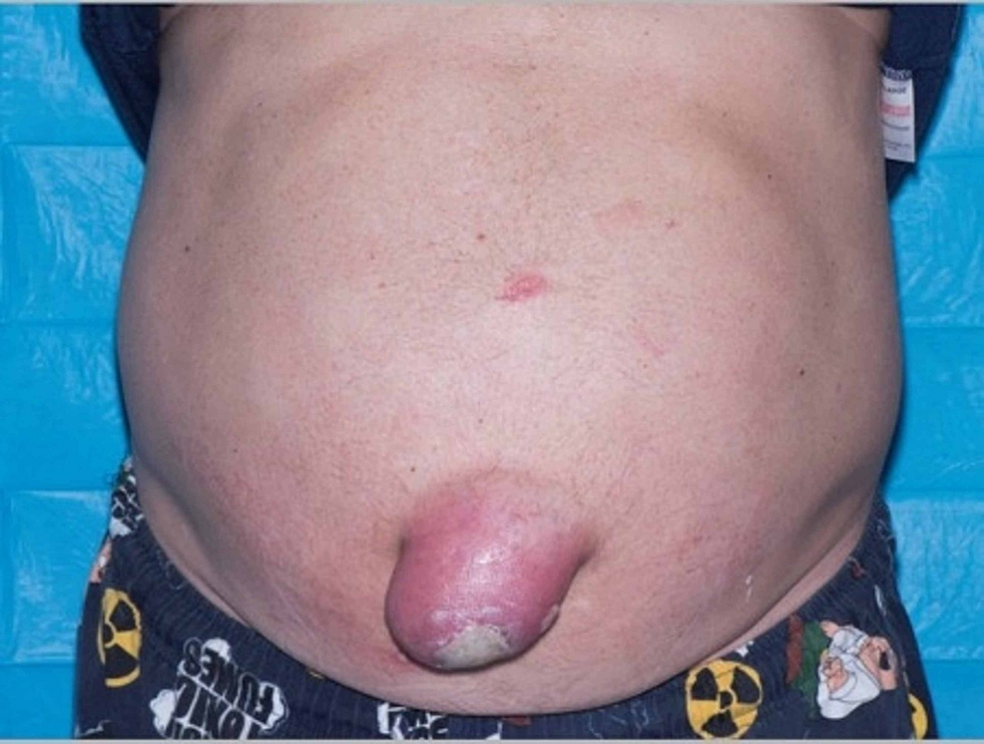
Umbilical hernia showing the area of full-thickness burn.

**Figure 2:**
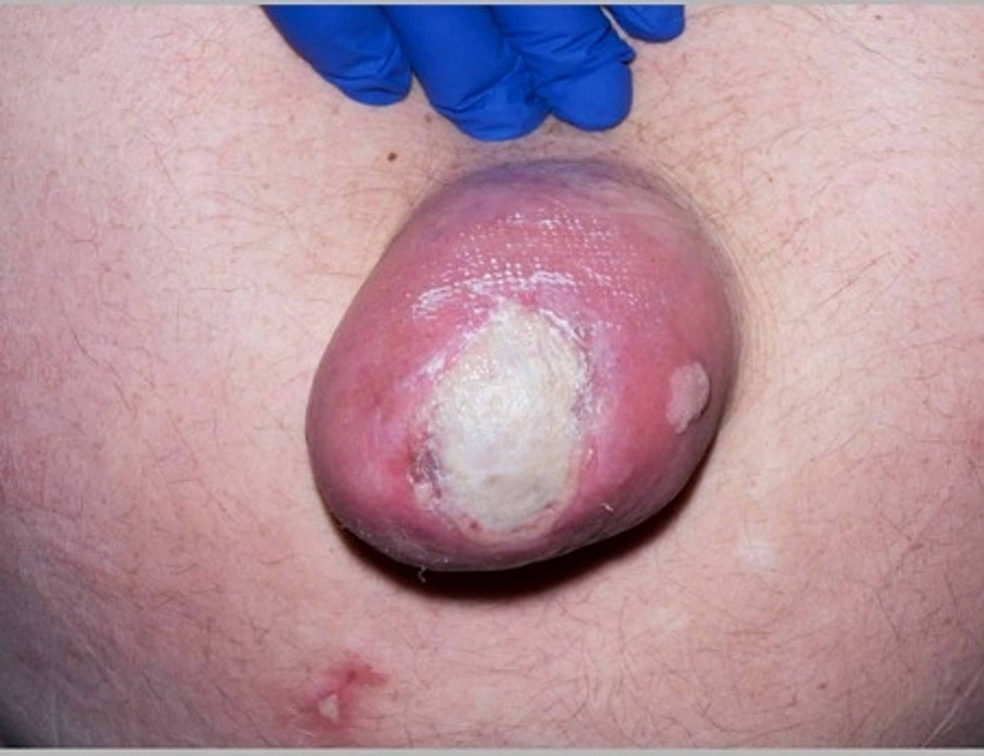
Umbilical hernia with full-thickness burn showing surrounding cellulitis.

At the presentation in the burns centre, the patient was systemically well with no abdominal symptoms and normal bowel motions. On examination, he had a 0.25% full-thickness burn (Figure 1 and Figure 2) with surrounding cellulitis supported by raised biochemical markers of inflammation; a C-reactive protein of 21mg/L and WCC of 11x10^9^/L. Wound swabs from the burn site later confirmed the presence of Staphylococcus aureus sensitive to flucloxacillin. Empirical therapy of flucloxacillin was already commenced intravenously at the time of presentation; 2g four times a day. Subsequent repeat blood tests showed a response to antibiotic therapy with a reduced C-reactive protein of 14mg/L and WCC of 8.6x10^9^/L on day 6 of admission.

The abdomen was soft and non-tender with bowel sounds present throughout. The general surgical team was asked to review the patient on day one of the presentation and a decision was made to perform a CT abdomen to determine both the underlying anatomy of the hernia sac, its contents and whether the full thickness burn was communicating with any loops of bowel. The CT scan demonstrated an umbilical hernia with a neck of 2.3cm. There was a breach in the hernia fascia, but no communication between bowel and the burn. The general surgical opinion was to aim for definitive surgical repair of the hernia with a mesh.

Optimal management of a full-thickness burn would typically be surgical debridement of the area to remove burnt and infected tissue. However in this case there was concern that surgical excision could potentially lead to the spread of infection deeper into the peritoneal cavity. The active infection would also be a contraindication for the use of mesh to repair the hernia.

After extensive discussion between the plastic surgery and general surgery teams, it was agreed upon to manage the burn non-operatively and allow the cellulitis to resolve before considering hernia repair. The full-thickness burn was managed with daily flamazine, jelonet, gauze and hypafix dressings. This dressing allowed the eschar of the burn to gently lift off and aid healing underneath with further epithelialisation. The patient was trained by the nurse specialists as to how to change his dressings himself at home. He was discharged with follow-up outpatient consultations to review the wound three times per week for the next two weeks. 

A few weeks later, the patient was reviewed by the general surgery team. The decision at this outpatient appointment was to proceed with elective hernia repair with a mesh as his burn had reduced in size and the acute infection had been treated. 

## Discussion

Recent trends in burns injuries show a positive outcome with a global reduction of burns incidence and severity [2]. This could be attributed to specialised care being centralised, burn prevention programmes and more advanced therapies. Despite these great paradigm shifts in the care of burns some inequalities still persist. An American study identified links of burn injuries with gender, occupation and socio-economic class; males in manual labour industries had a higher frequency of burns-related injury as in the case described in this report [3].

Upon review of the current literature, we found two paediatric cases describing burns to the skin over an umbilical hernia. In 1980, Mungas and Reyes reported a burn complication in a three-month-old patient, who developed perforated small bowel following a burn to an umbilical hernia [4]. This case report refers to an older report by Boswick and Raffensperger in which a 5-month-old female also experienced perforated small bowel from an umbilical hernia burn [5]. Our case report is the first adult case of an umbilical hernia burn reported in the literature.

These paediatric case reports both described more severe clinical sequelae following the injury. It was hypothesised that this could be attributed to differences in the skin of infants and adults. Oranges et al. refer to the difference in skin architecture and function, with impaired barrier functionality and a thinner epidermis in neonates making them more susceptible to full-thickness burns [6]. The same level of thermal or chemical burn insult in a neonate would result in a more severe burn than compared to an adult. In our case report, this anatomical difference could account for why a full-thickness burn was sustained without small bowel perforation. In addition, the hernial sac contained omental fat rather than small bowel which also accounts for a better outcome from the burn injury.

With regards to the surgical management of both the burn and the hernia, the complicating factor, in this case, was an acute infection at the burn site. Full-thickness burns result in a complete loss of the dermis and therefore leaves the wound bed with no epidermal cells to regenerate [7]. To manage this, grafted skin is often used to replace the lack of regenerating cells and heal the wound by a process of graft take. Excising a full-thickness burn also removes the non-viable tissue and reduces the infection risk. In our case, the patient already had surrounding burn cellulitis at the time of admission highlighting the need for surgical management. However due to the unusual anatomical site of injury, with such proximity to bowel in the hernia, determining how best to treat the full thickness burn and reduce the infective burden was a challenge. 

Bueno-Lledo et al. experienced a 1.9% (n=66) infection rate in 3470 cases of abdominal wall hernia repair with mesh [8]. This was due to risk factors such as steroid use, immunosuppressants and surgical site infection. In this case, of a diabetic patient where the surgical site was already infected, the potential increased risk of mesh infection had to be taken into account when deciding the best course of further management. After extensive discussion with our general surgical colleagues, a plan was developed to manage the full thickness burn conservatively, treat the acute infection, and then perform an elective hernia repair at a later date.

## Conclusions

A full-thickness burn over an umbilical hernia is an extremely rare injury. This case highlights the importance of taking anatomical considerations into account when planning whether surgical excision and debridement of the damaged tissue are appropriate or not. It demonstrates that, in certain cases, patients with full-thickness burns can be managed effectively with conservative treatment and frequent clinic reviews to monitor progress. A co-ordinated multi-disciplinary team approach is essential in the treatment of burns and enabled the development of an optimal management plan for this patient.
